# Repetitive Short-Term Stimuli Imposed in Poor Mixing Zones Induce Long-Term Adaptation of *E. coli* Cultures in Large-Scale Bioreactors: Experimental Evidence and Mathematical Model

**DOI:** 10.3389/fmicb.2017.01195

**Published:** 2017-06-28

**Authors:** Alexander Nieß, Michael Löffler, Joana D. Simen, Ralf Takors

**Affiliations:** Institute of Biochemical Engineering, University of StuttgartStuttgart, Germany

**Keywords:** scale-down, hybrid modeling, population heterogeneity, adaptation times, *Escherichia coli*

## Abstract

Rapidly changing concentrations of substrates frequently occur during large-scale microbial cultivations. These changing conditions, caused by large mixing times, result in a heterogeneous population distribution. Here, we present a powerful and efficient modeling approach to predict the influence of varying substrate levels on the transcriptional and translational response of the cell. This approach consists of two parts, a single-cell model to describe transcription and translation for an exemplary operon (*trp* operon) and a second part to characterize cell distribution during the experimental setup. Combination of both models enables prediction of transcriptional patterns for the whole population. In summary, the resulting model is not only able to anticipate the experimentally observed short-term and long-term transcriptional response, it further allows envision of altered protein levels. Our model shows that locally induced stress responses propagate throughout the bioreactor, resulting in temporal, and spatial population heterogeneity. Stress induced transcriptional response leads to a new population steady-state shortly after imposing fluctuating substrate conditions. In contrast, the protein levels take more than 10 h to achieve steady-state conditions.

## Introduction

Large-scale industrial bioprocesses make use of reactors ranging from 100 to 800 m³ reaction volume. For aerobic processes, stirred tank reactors are still preferred, albeit alternative setups such as airlift reactors may be attractive if reactor sizes exceed the volume of about 500 m³. All reactors have in common that gradients of substrates, dissolved gases and pH occur, which are the consequence of poor mixing conditions (Nienow et al., 1997). Cells are circulating in these reactors, thereby frequently passing through zones of different substrate availability. Accordingly, cellular interactions are repeatedly triggered (Oldiges and Takors, 2005; Lara et al., 2006; Neubauer and Junne, 2010; Takors, 2012). Noteworthy, related regulatory responses are not limited to changes of metabolism but also comprise transcriptional and translational programs (Löffler et al., 2016, 2017; Simen et al., 2017).

Often, microbial processes are controlled by limited substrate feeding to avoid non-wanted overflow metabolism and to prevent too high metabolic activity that may exceed the technical capacities of aeration and cooling. Industrial examples are the implementation of glucose or ammonia limitations (Neubauer et al., 1995). Recently, Chubukov et al. (2014) outlined that proper nitrogen (or phosphate) limitation may even increase biomass specific substrate uptake during production phases when cell growth is strongly limited. Michalowski et al. (2017) further succeeded to engineer the *E. coli* HGT host for likewise conditions.

Löffler et al. (2016) and Simen et al. (2017) studied the scenario of frequently occurring glucose or ammonia limitations by using a conventional STR-PFR (stirred tank reactor—plug flow reactor) setup as described by George et al. (1993). Unlike previous investigations, these studies installed steady-state growth conditions before large-scale gradients were repeatedly imposed on the cells by connecting the PFR to the STR. As such, a distinct reference steady-state was created that enabled quantitative and highly accurate analysis of the metabolic and transcriptional responses of the cells on the installed glucose or ammonia gradients.

These data sets are the experimental basis for the modeling approaches presented in this study. By exploiting the metabolic and transcriptional time series it will be investigated whether and how similar dynamics can be modeled to predict short- and long-term regulatory responses of *E. coli*. Related data-driven models can serve as the core for ensemble modeling (Henson, 2003) to predict large scale cellular performance *in silico* and *ab initio*.

For the sake of simplicity, transcriptional dynamics of the tryptophan operon were chosen as an illustrative example. It has been shown by Simen et al. (2017) that the repetitive exposure to nitrogen starvation induced the frequent transcription of the *trp* operon. Considering the well-known attenuation control (Yanofsky, 2004, 2007), the expression of downstream genes *trpEDCBA*, consequently, indicates not only the ongoing transcriptional response on environmental triggers but also the start of protein translation. Accordingly, modeling *trp* expression dynamics needs to fulfill several challenges: (i) Short-term transcript dynamics observed in the PFR must be predicted, (ii) long-term transcript responses of the whole population should be mirrored, and (iii) the different time-scales of transcriptional and translational dynamics have to be reflected, too. This study will outline that every constraint is properly met by a simple mechanistic model.

## Materials and methods

### Experimental setup

**Figure 1:**
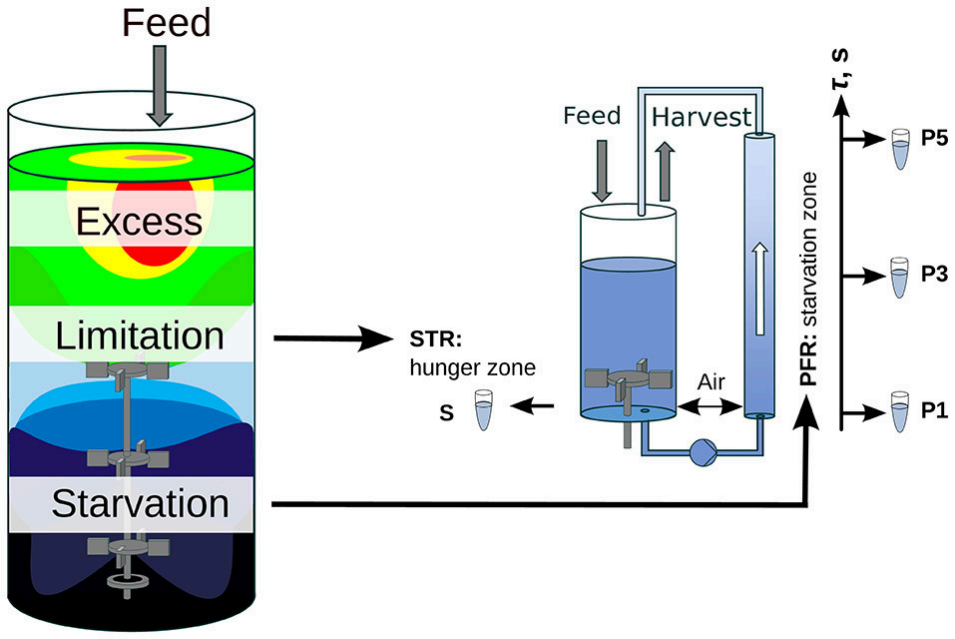
Scheme of the scale-down approach. A large-scale bioreactor and its substrate gradient is simulated by a chemostat STR (limitation zone) coupled to a PFR (starvation zone). This approach allows the examination of the influence of substrate gradients (i.e., glucose or ammonia) on the population dynamic.

Oscillating substrate availability was simulated in a stirred-tank-reactor (STR) plug-flow-reactor (PFR) scale-down approach. Figure 1 shows the schematic setup of the system. As Simen et al. (2017), the STR system was operated as nitrogen limited chemostat cultivation with a dilution rate of 0.2 h^-1^ (5 mL min^-1^). The well-mixed bioreactor was simulated by using the steady-state condition in STR without the PFR (SS_0_). After characterization of SS_0_ the PFR was connected and a fraction of cells cycled through the PFR loop. No additional feed was added into the PFR. Therefore, the cells shift from nitrogen limitation to starvation along the PFR. The experimental design allows the observation of the transcriptional responses along the PFR (short-term) and over the process time in the STR (long-term). The system characteristics and cultivation conditions were published in Löffler et al. (2016). Residence times τ of STR and PFR were estimated to be 6.2 min and 125 s, respectively. Samples for transcriptome analysis were taken at sampling ports P1, P3, and P5 in the PFR with corresponding residence times of 31, 70, and 110 s. Volumes in STR and PFR were 1.12 and 0.38 L, respectively. Biosuspension was continuously pumped through the PFR (180 mL min^-1^).

Compared to SS_0_ with a growth rate of 0.2 h^-1^ in the STR, the STR-PFR setup splits growth rates individually in the two compartments. Whereas, the total growth rate of the STR-PFR system is still 0.2 h^-1^, no growth can be expected inside PFR when the nutrient is completely consumed. Accordingly, PFR can be subdivided into a first part considering still growing cells and a subsequent part characterized by no growth. Because the total system runs with the dilution rate of 0.2 h^-1^, STR growth rates must be increased accordingly to compensate missing cell growth in the PFR. Calculation of growth rates in the STR can be performed based on the residence time distributed average growth rate that is set equal to the dilution rate. For the sake of simplicity and because nutrient starvation occurred very rapid in PFR, no growth was assumed to be existent in the total PFR compartment. Therefore, the average growth rate can be split into two different growth rates for each compartment (Equation 1).

(1) $$\frac{\mu_{STR}\;\tau_{STR}+\mu_{PFR}\tau_{PFR}}{\tau_{STR}+\tau_{PFR}}=D$$

Samples for transcriptome analysis were taken at 25 and 120 min as well as at 28 h after installing the substrate gradient. Transcript measurements are published in Simen et al. (2017) and are available under GEO Accession GSE90743.

### Single-cell model

For the agent based transcription-translation model, DNA and mRNA templates are discretized in nucleotides, defining a 1D lattice. Movement of RNA polymerases (RNAPs) is treated according to Equation (2). Here, *x* describes the relative position of nucleotides inside the operon starting with the first mRNA encoding nucleotide of the operon sequence. RNAP movement is based on the elongation rate $v_{elo}^{RNAP}$ and the distance Δ*x* between two subsequent polymerases. The following criteria were considered for RNAP motion:

- The first elongation step is treated as the initiation step and can only occur if *t* is in the interval of possible induction (*t*_*ind*_).
- The minimum distance Δx between two subsequent RNAPs is fulfilled.

(2) $$\frac{dx_{i}}{dt}=\{\begin{array}{c} 0\\ 0\\ v_{elo}^{RNAP} \end{array}\;\begin{array}{c} if\;x_{i}=0\;\wedge \;t\notin t_{ind}\\ if\;x_{i-1}-x_{i}<\Delta x\;\wedge i>1\\ otherwise \end{array}$$

For each passed nucleotide on the DNA sequence, the respective nucleotide in the mRNA sequence is transcribed. The resulting mRNA strand *i* with length $L_{i}^{mRNA}$ can be directly derived from *x*_i_.

(3) $$L_{i}^{mRNA}\left(t\right)=x_{i}\left(t\right)$$

For simplification, we neglected the modeling of the attenuation process considering terminator/antiterminator interactions and assumed ongoing translation only during nitrogen starvation instead. Position *y*_i, j_ of a ribosome *j* on mRNA strand *i* is a function of $L_{i}^{mRNA}$ and the position of the previous ribosome *y*_*j*−1_. The number of ribosomes that translate a gene *g* ($N_{g}^{TL,\max}$) can vary and depends on the gene itself. Ribosomal motion on a gene *g* starts at $C_{g}^{start}$ (first coding nucleotide) and stops at $C_{g}^{end}$ (third nucleotide of the terminating codon). The necessary criteria for translation are stated as follows:

- At least Δy nucleotides downstream are already synthesized.
- The previous ribosome is more than Δy nucleotides further downstream.
- The maximum number of translations for the given gene is not exceeded.

(4) $$\frac{dy_{i,j}}{dt}=\{\begin{array}{c} 0\\ 0\\ 0\\ v_{elo}^{Ribosome} \end{array}\;\begin{array}{c} if\;L_{i}-y_{i,j}\leq \Delta y\\ if\;y_{i,j-1}-y_{i,j}<\Delta y\;\wedge j>1\\ \begin{array}{c} N_{i,g}^{TL}\left(t\right)\geq N_{g}^{TL,\max}\\ otherwise \end{array} \end{array}$$

The number of ribosomes acting on a single mRNA *i* is calculated following the Iverson brackets (Equation 5). These brackets return 1 if the term inside is true and 0 if the term is false.

(5) $$N_{i,g}^{TL}\left(t\right)=\sum_{j}\left[y_{i,j}\left(t\right)\geq C_{g}^{start}\right]$$

The amount of synthesized proteins per cell from the single-cell model ($N_{g,\;SC}^{Protein}$) encoded by gene *g* can be calculated as the sum of all ribosomes acting on all mRNA strands that have passed the final nucleotide $C_{g}^{end}$.

(6) $$N_{g,\;SC}^{Protein}\left(t\right)=\sum_{i}\sum_{j}\left[y_{i,j}>C_{g}^{end}\right]$$

Each mRNA strand is expected to be degraded by RNases. Initiation of mRNA breakdown begins at the start codon of transcription. Movement along mRNA is encoded by position *z*_i_ on strand *i* and depends on the degradation elongation rate $v_{elo}^{RNAse}$. The following constraints define the motion of RNAses:

- The number of active ribosomes per gene g $N_{g}^{TL,\max}$ is estimated as the turnover ratio of mRNAs and proteins (see below)
- Δz encodes the closest nucleotide distance to the next ribosome downstream of *z*_i_

(7) $$\frac{dz_{i}}{dt}=\{\begin{array}{c} 0\\ 0\\ v_{elo}^{RNase} \end{array}\;\begin{array}{c} if\;N_{i,g}^{TL}\left(t\right)<N_{g}^{TL,max}\;\\ if\;y_{i,\;N_{g}^{TL,max}}-z_{i}\leq \Delta z\\ otherwise \end{array}$$

Accordingly, the current amount of mRNA per gene is calculated as the difference of already synthesized mRNAs and the amount of degraded mRNAs. The first is modeled from the number of complete mRNA strands synthesized. The second mirrors the amount of RNases that have passed the first codon.

(8) $$N_{g,\;SC}^{mRNA}\left(t\right)=\sum_{i}\left[L_{i}^{mRNA}\left(t\right)>C_{g}^{end}\right]-\sum_{i}\left[z_{i}\geq C_{g}^{start}\right]$$

$N_{g}^{TL,\max}$ is calculated as the turnover ratio of mRNAs per protein for a given gene *g*. Protein turnover $r_{turnover}^{Protein}$ was calculated based on protein levels at a growth rate of 0.2 h^-1^ ($k_{\deg}^{Protein}=\mu$) (Valgepea et al., 2013). Active protein degradation was neglected and only growth based dilution was considered. mRNA turnover $r_{turnover}^{mRNA}$ was calculated based on the levels measured by Valgepea et al. (2013) with average half-lives of 2 min ($k_{\deg}^{mRNA}=20.79\;h^{-1}$) (Chen et al., 2015). However, no mRNA measurements of *trpA* were given in this data set. We thus assumed the translations per mRNA for *trpA* to be the same as for *trpB*, due to the fact that the resulting protein complex is a tetramer consisting of two *trpA* and two *trpB* (Hyde et al., 1988). *TrpL*, the leader peptide, was neglected in this calculation. Table 1 shows the resulting translations per mRNA.

(9) $$N_{g}^{TL.\max}=\frac{r_{turnover}^{Protein}}{r_{turnover}^{mRNA}}=\frac{c_{g}^{Protein}\;k_{\deg}^{Protein}}{c_{g}^{mRNA}\;k_{\deg}^{mRNA}}$$

**Table 1:** Calculated translations per mRNA for the *trp* operon.

**Gene**	***trpE***	***trpD***	***trpC***	***trpB***	***trpA***
Translations per mRNA	4	4	5	10	10

*The value for trpA was extrapolated from trpB*.

**Figure 2:**
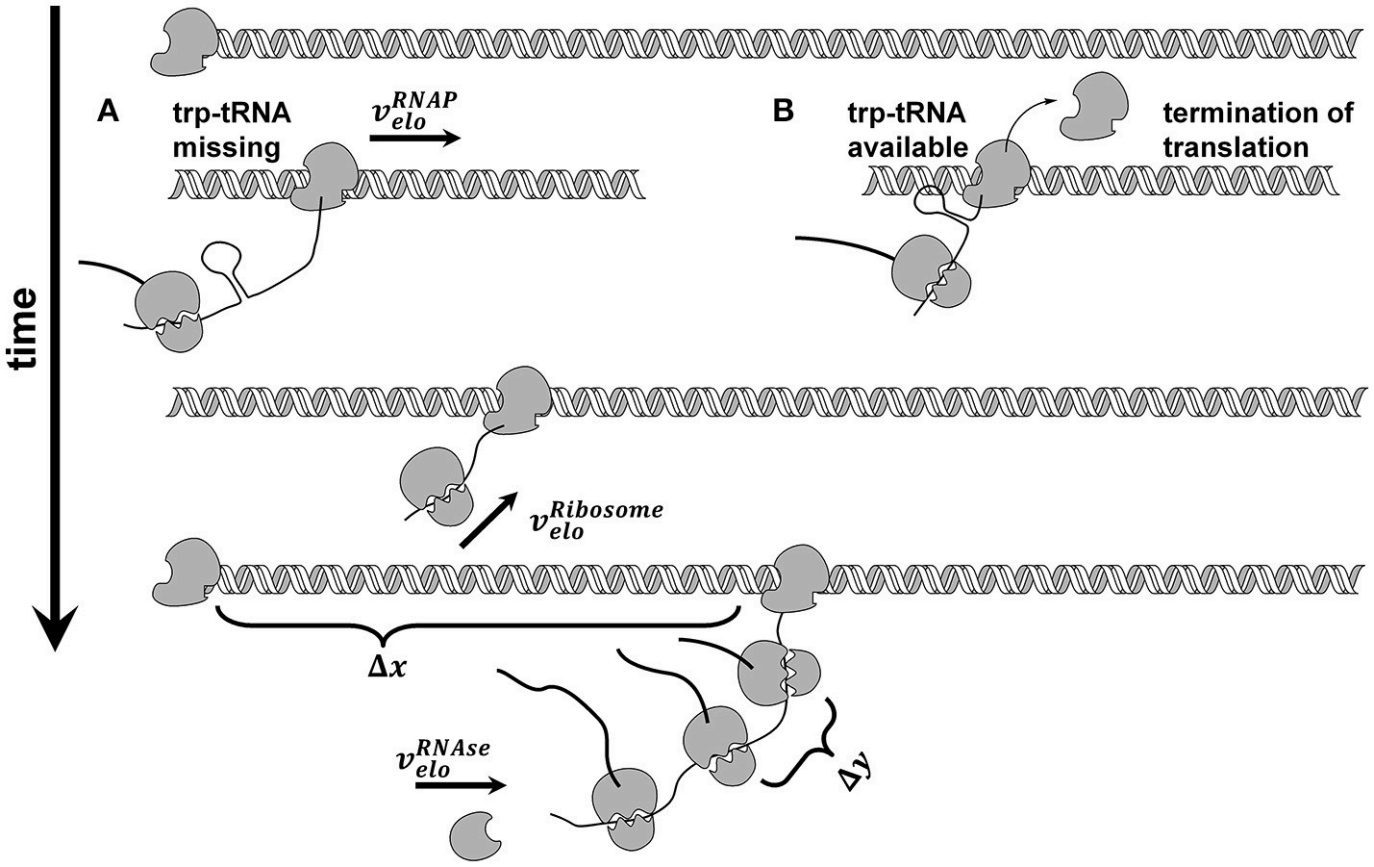
Model of coupled transcription and translation. RNA polymerase (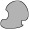) binds to the *trp* promotor and starts transcribing with constant elongation rate. After transcription of each genes ribosome binding site, translation takes place and ribosomes (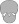) elongate with constant elongation rate that is set equal to RNAP rate. If trp-tRNA is missing, translation continues **(A)**, if trp-tRNA is available, a terminator sequence forms and translation stops **(B)**. Each gene has its own number of translations before degradation takes place. Degradation by RNases (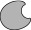) starts at the 5′ end and continues from gene to gene if the mentioned number of translations already took place.

We used the *trp* operon as an example for several reasons: (i) The *trp* operon leads to a polycistronic mRNA (Yanofsky et al., 1981), (ii) the attenuation sequence in the *trpL* leader peptide allows the coupled investigation of transcription and translation (see Figure 2), and (iii) the published data by Simen et al. shows that the *trp* operon is upregulated during STR-PFR cultivations. Accordingly, translation must have happened if transcripts of genes downstream of *trpL* are measured, as it is the case in the data sets used for this study. Simplification was made by treating the structural genes *trpGD* and *trpCF* and their corresponding proteins as single genes (*trpG* and *trpC*, respectively) and proteins.

All three actively moving species (RNAP, ribosomes and RNase) are treated as equally fast and their elongation rate was taken from the RNAP elongation rate reported by Chen et al. (2015) and set to 21 nucleotides s^-1^ (see Table 2). Minimum distances Δ *x*, Δ *y*, and Δ *z* were set to 100 nucleotides each [which is larger than (Bremer and Dennis, 1987) estimated for a growth rate of 0.5 h^-1^].

**Table 2:** Model parameters used for simulation of both single-cell and cell distribution model.

**Parameter**	**Value**	**Unit**
$\nu_{elo}^{RNAP}$	21	Nucleotides per second
$\nu_{elo}^{Ribosome}$	21	Nucleotides per second
$\nu_{elo}^{RNase}$	21	Nucleotides per second
Δx	100	Nucleotides
Δy	100	Nucleotides
Δz	100	Nucleotides
*t*_*ind*_	[30 125]	Seconds
$\dot{\text{V}}$_*PFR*_	180	mL min^-1^
$\dot{\text{V}}$_*Feed*_	5	mL min^-1^
V_*STR*_	1,120	mL
*D*	0.2	h^-1^
$N_{STR}^{0}$	10,000	cells

Each PFR passage induced transcription, however, with a delay of 30 s based on experimental observations. Once induction has started and RNAP has passed the attenuation sequence, transcription was assumed to continue until the terminator sequence after *trpA* was reached (see Figure 2).

### Cell distribution model

The ensemble cell model needs to be embedded in a process model for describing the flow wiring and residence times of the cells in the compartments. The PFR is considered as a plug flow reactor showing almost equally distributed residence times for all cells. The STR is assumed to be ideally mixed, thus, having a residence time distribution constrained by the reaction volume and the throughput. Dilution and growth rate additionally influence the population.

For population balancing, the following events were considered to track the fate of each individual cell:

Cells may

1. leave the STR for entering the PFR and cycle back into STR after τ_*PFR*_, the residence time in the PFR
2. be drained off by the efflux (harvest)
3. divide, setting all transcriptional and translational programs on default (no initiation of transcription or translation in the corresponding daughter cell)

The following probability functions α_*i*_ were defined

(10) $$\alpha_{1}=N_{STR}\frac{{\dot{V}}_{PFR}}{V_{STR}}$$

(11) $$\alpha_{2}=N_{STR}\frac{{\dot{V}}_{Feed}}{V_{STR}}$$

(12) $$\alpha_{3}=N_{STR}^{0}\;D$$

For modeling event (1), the rate α_1_is used, indicating that a cell leaves the STR and enters the PFR again. Washout of cells (event 2) was treated equally with the dilution rate as flux value (α_2_). The probability for cell division (α_3_) is based on the set dilution rate *D* and the cell number $N_{STR}^{0}$ during SS_0_. Return of cells from the PFR compartment was fixed to occur after τ_*PFR*_ passed. Cells that are washed out by event (2) are deleted from the system and newly born cells from event (3) are treated as default daughter cells without any transcriptional deflection.

The reaction system was numerically solved by applying Gillespie's stochastic simulation algorithm (Gillespie, 1977). Time increment τ was solved based on the sum of the three reaction events considering the probabilities as indicated in Equation (13). The chosen reaction *i* is calculated, based on Equation (14). *r*_1_ and *r*_2_ are uniformly distributed random variables in the interval (0, 1).

(13) $$\tau =\frac{1}{\sum \alpha_{i}}\ln\left(\frac{1}{r_{1}}\right)$$

(14) $$\sum_{j\;=1}^{i-1}\alpha_{j}\leq r_{2}\sum_{j\;=1}^{3}\alpha_{j}\leq \sum_{j\;=1}^{i}\alpha_{j}$$

Simulations were performed using 10,000 cells, assuming uniform distribution in the STR ($N_{STR}^{0}$) before it is connected to the PFR. Simulations tracked cell numbers in the STR and the PFR as well as each transition of a cell from STR to PFR.

### Coupling of single-cell and cell distribution model

To minimize computational efforts, the impact of single-cell metabolic activities on the local environment was ignored. In essence, cells were considered to travel through a “frozen” bioreactor background that triggers transcriptional and translational responses as reflected in the single-cell model. For balancing the population distribution properly, the history of every cell was tracked. As the trigger “PFR” is of outstanding importance, the entrance of each cell into the PFR was logged. The resulting in a set of time flags ($t_{i}^{flag}$) for each cell that was stored for the total simulation period, which allows detailed tracking of the cells motion in the STR-PFR setup. Additionally, the events (2) and (3) were tracked for each cell allowing the calculation of the population distribution in the STR and the PFR at each time step of simulation.

The simulation approach allowed the independent solution of the single-cell and cell distribution model. Simulations of the single-cell model resulted in distinct mRNA ($N_{g,\;SC}^{mRNA}\left(t\right)$) and protein ($N_{g,\;SC}^{Protein}\left(t\right)$) patterns for every cell entering and leaving the PFR and this constant sequence can be stored as look-up table. In the distribution model, each flag indicates start of induction, whose sequence is stored in the look-up table. Duration of an induction phase is defined from entering the PFR at *t*^*flag*^ until the last mRNA is degraded at *t*^*flag*^+Δ *t*. Superposition of all transcriptional and translational patterns over the cells lifetime results in a continuous description of transcriptional and translational patterns in the STR-PFR system.

Cellular growth by event (3) is treated as generation of a new default cell without any additional mRNA and protein content without altering the mother cell.

(15) $$N_{g}^{mRNA}\left(t\right)=\sum_{i}\{\begin{array}{c} N_{g,SC}^{mRNA}\left(t-t_{i}^{flag}\right)\\ 0 \end{array}\;\begin{array}{c} if\;t-t_{i}^{flag}\leq \Delta t\\ otherwise \end{array}$$

(16) $$N_{g}^{Protein}\left(t\right)=\sum_{i}\{\begin{array}{c} N_{g,SC}^{Protein}\left(t-t_{i}^{flag}\right)\\ N_{g,SC}^{Protein}\left(\Delta t\right) \end{array}\;\begin{array}{c} t-t_{i}^{flag}\leq \Delta t\\ otherwise \end{array}$$

## Results

### Key assumptions

Löffler et al. (2016) and Simen et al. (2017) showed that the repeated oscillation of the substrate availability of *E. coli*, simulated with a STR-PFR system, induce repeated on/off switching of several hundred genes. Among them, the frequent activation of the tryptophan operon could be observed (Simen et al., 2017). The mathematical model comprising the (Equations 2–16) was used to describe not only short- and long-term transcriptional dynamics but also to estimate the impact on protein formation by linking the transcription with the translation machinery. The following key assumptions were made: (i) Once transcription of mRNA has started, it continued until the stop signal was achieved at the end of the operon, namely on the relative position 6726 nt after *trpEDCBA* (Stoltzfus et al., 1988), (ii) mRNA was assumed to be immediately translated into proteins. The number of active ribosomes per gene (encoding mRNA) was calculated based on the experimental findings of Valgepea et al. (2013).

### Modeling short-term transcriptional dynamics

**Figure 3:**
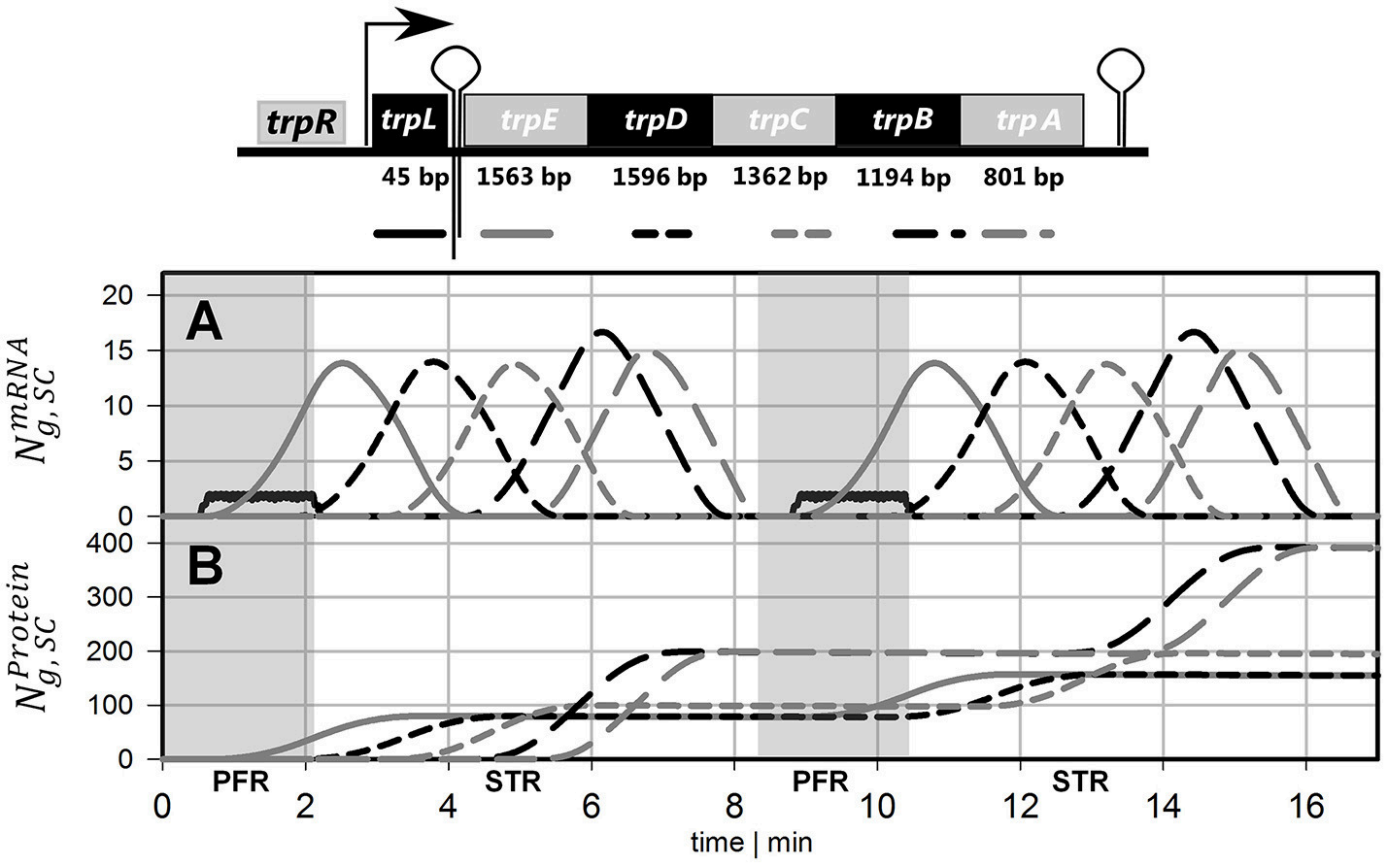
Time courses of two subsequent cell cycles comprising PFR and STR passage. **(A)** mRNA and **(B)** protein profiles are shown, both simulated using the single-cell model. The genes and their gene products are encoded as follows: Black solid line = *trpL*, gray solid line = *trpE*, black short dashed line = *trpD*, gray short dashed line = *trpC*, black long dashed line = *trpB*, gray long dashed line = *trpA*. Gray shaded areas indicate that the cell currently passes the PFR.

The simulation of transcriptional dynamics during a single PFR passage was achieved using the single-cell model with the parameters listed in Table 1. Figure 3A depicts mRNA courses of two subsequent PFR-STR passages. At *t* = 0, the PFR entering cell is induced and initiates transcription after the experimentally observed delay time of 30 s. Then, transcription of the *trp* operon starts with *trpL*. As shown, *trpL* is fully and *trpE* partially transcribed before the cell leaves the PFR. Accordingly, the remaining genes downstream of the operon were transcribed after the cell reenters the STR. Shortly after initiation, degradation of *trpL* mRNA has started, as indicated by the constant mRNA levels. After leaving the PFR, the cell stops further RNAP initiation and RNases immediately degrade the remaining transcripts. Noteworthy, all gene transcripts were fully degraded (except for a small residual of *trpA*) before the cells again reentered the PFR.

### Modeling protein formation

Based on the single-cell model, translation of mRNA was simulated as depicted in Figure 3B. It was assumed that protein formation started as soon as the ribosomal binding site was transcribed. Because *trpL* encodes the leader peptide, translation modeling was simply focused on *trpEDCBA*. First, TrpE proteins were produced while the cells passed the PFR compartment. Downstream proteins were translated after the cells reentered the STR. Consequently, the majority of translation happened in STR. Protein formation is delayed and multiplexed compared to mRNA production. Accordingly, dynamics of protein courses are less steep than those of transcript levels. The latter are characterized by fast transcription and fast mRNA degradation that finally lead to sharp peaks of transcript contents. Protein degradation is slower by orders of magnitude. Consequently, only moderate pool dynamics and even protein accumulation was observed after PFR-STR transits.

Each PFR-STR cycle lasted for about 500 s. During this period, cells managed to produce 20 mRNA copies of the complete *trp* operon. Subsequent translation enabled the formation of 80 TrpED, 100 TrpC, and 200 TrpBA copies (considering the ribosomal stoichiometry of Table 1) with corresponding translation rates of 9.6, 12 and 24 proteins per cell per minute.

As outlined above, protein degradation is known to be much slower than mRNA decay which enabled the simplified simulation of protein dynamics shown in Figure 3B. However, the scenario may change if steady-states are analyzed. As outlined in equation 17, steady-state protein levels will be dependent on the degradation constant.

(17) $$\begin{array}{l} \frac{dc_{Protein}}{dt}=r_{Translation}-r_{\deg}=r_{Translation}\\ \;-c_{Protein}\;k_{\deg}^{Protein}=0 \end{array}$$

(18) $$c_{Protein}=\frac{r_{Translation}}{k_{\deg}^{Protein}}$$

**Figure 4:**
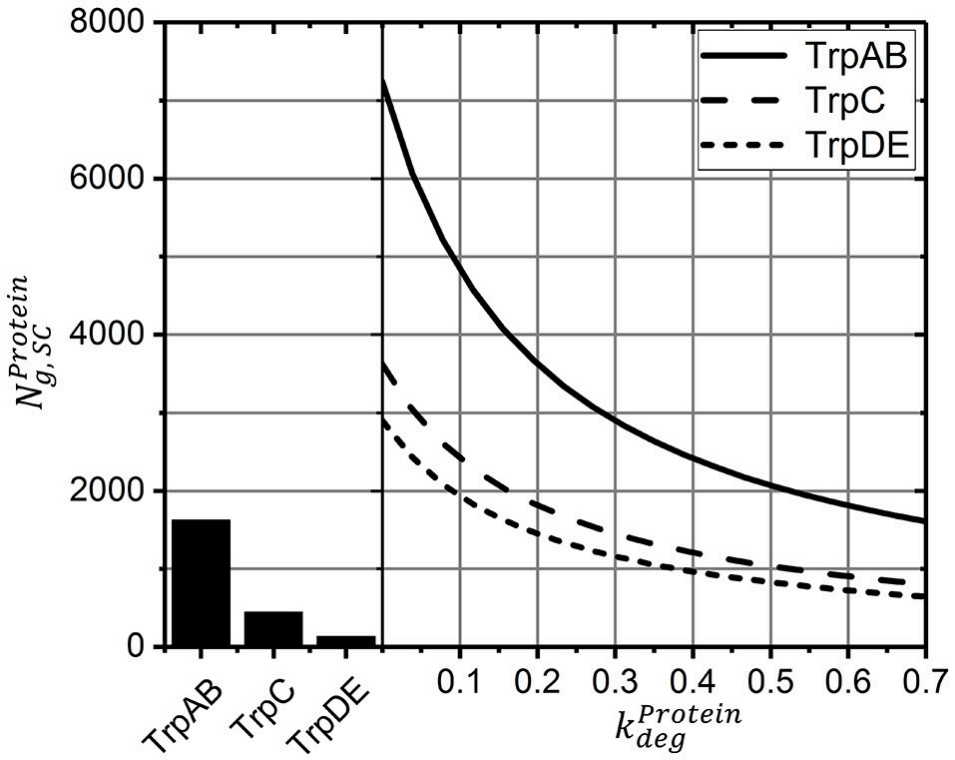
Protein levels for the new steady-state after >15 h as a function of the degradation constant (solid lines). Higher values show the influence of declining half-lives and thus higher degradation constants. Measured protein levels (bar graph) were taken from Valgepea et al. (2013).

Because the individual degradation constants for the *trp* gene products are unknown, simulation studies were performed and summarized in Figure 4. In essence, results for $k_{\deg}^{Protein}=0$ indicate protein loss simply based on cell drain under continuous operating conditions whereas results with $k_{\deg}^{Protein}>0$ consider additional protein degradation with the given rates. For comparison, experimental results are indicated, too. As shown, when $k_{\deg}^{Protein}$ exceeds 0.6 h^-1^ (which corresponds to half-lives lower than 1.1 h) simulated protein levels are smaller than those reported for the given growth rate of 0.2 h^-1^. Accordingly, the simplifying assumption to neglect protein degradation for simulating STR-PFR dynamics is validated as half-lives of 1.1 h fairly exceed cycling times of about 500 s (about 0.12 h).

### Simulating long-term adaptation

As indicated in Löffler et al. (2016) and Simen et al. (2017), the STR-PFR experiments were performed as a continuous cultivation. First, glucose- or ammonia limited steady-states were installed cultivating the cells in STR only. Then, the PFR was connected while retaining the total system dilution rate of 0.2 h^-1^. As such, not only short-term transcript dynamics could be elucidated by sampling the PFR but also long-term adaptation of the whole population by studying transcript patterns in STR during the adaptation period of 28 h after PFR connection.

For simulation studies, the cell and the process model were linked predicting a stable distribution of 7526 ± 68 tracked cells in the STR (75.0 ± 0.68%) and 2513 ± 47 simulated cells in the PFR (25.0 ± 0.47%). Accordingly, the simulated cell population matched well with the volumetric setup comprising 74.7 vol% in the STR and 25.3 vol% in the PFR.

Neglecting the residence time distribution in the STR indicates that cells in the STR are always induced as shown in Figure 3. Therefore, population heterogeneity is not observable. Including residence time distribution for a perfectly mixed reactor reveals the existence of several subpopulations. Whilst 34% of the cells are currently not induced, 48% of the cells are currently induced once and 18% of the population are induced multiple times. Multiple inductions in this context indicate that the cell reenters the PFR while still being induced from a previous PFR passage, resulting in multiple transcription events (time dependency is shown in Supplementary Material).

**Figure 5:**
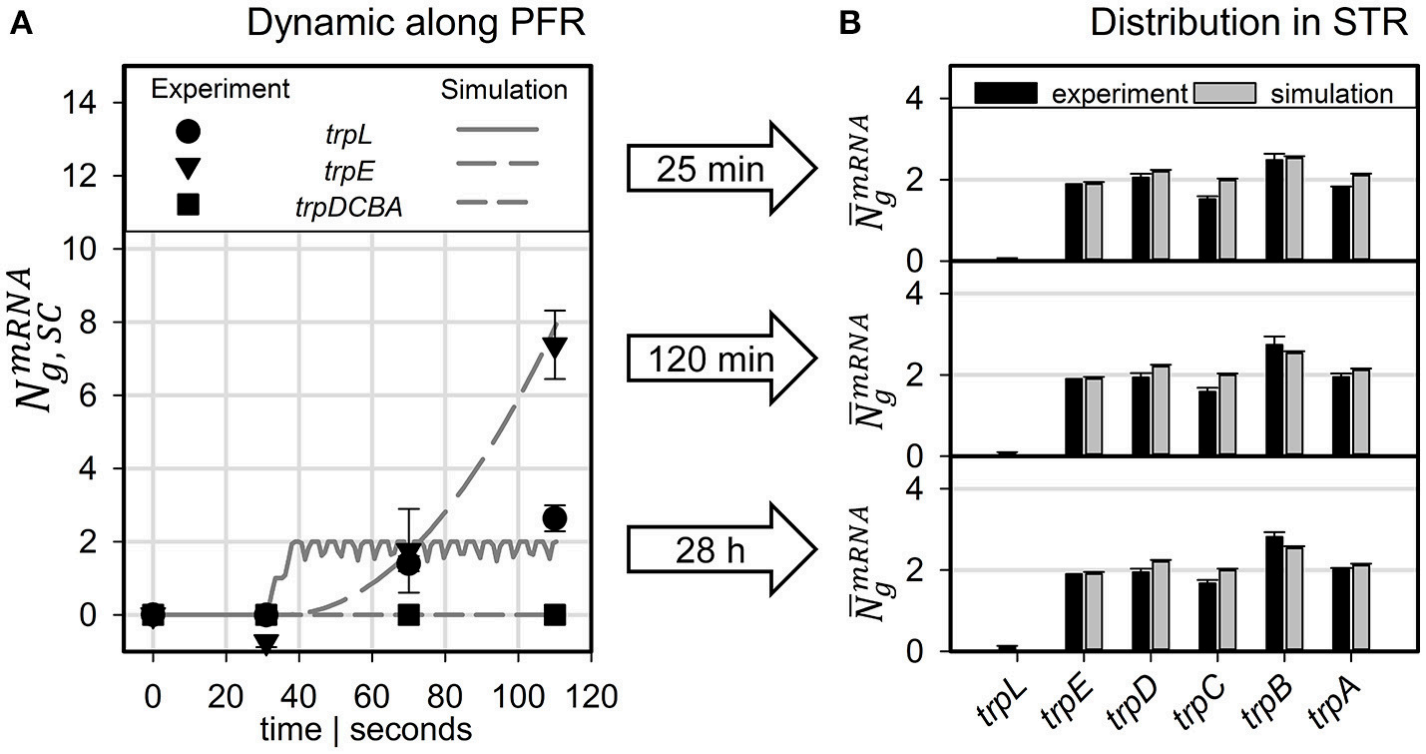
Predicted transcript levels for **(A)** PFR residence times of 30, 71, and 110 s compared to measured values (scaled to mean *trpLE* level) and **(B)** for the STR population compared to measured values (scaled to simulated *trpE* levels).

Figure 5A compares measured and simulated transcript dynamics of the *trp* operon while passaging through the PFR. Notably, measured transcript dynamics were very similar so that measurements taken after 25, 120 min and 28 h were cumulated and indicated by a common variance. According to the modeling constraints, mRNA production started after 30 s which is in good agreement with experimental observations for *trpL* and for *trpE*. Synthesis of further downstream genes *trpDCBA* was neither predicted nor measured.

The long-term adaptation of the population was simulated for the exemplary time points of 25 and 120 min as well as for 28 h (see Figure 5B). Again, experiments and simulation results show a high agreement for all conditions. This also holds true for the short *trpL* mRNA which was hardly detected in the PFR, confirming the simulation.

**Figure 6:**
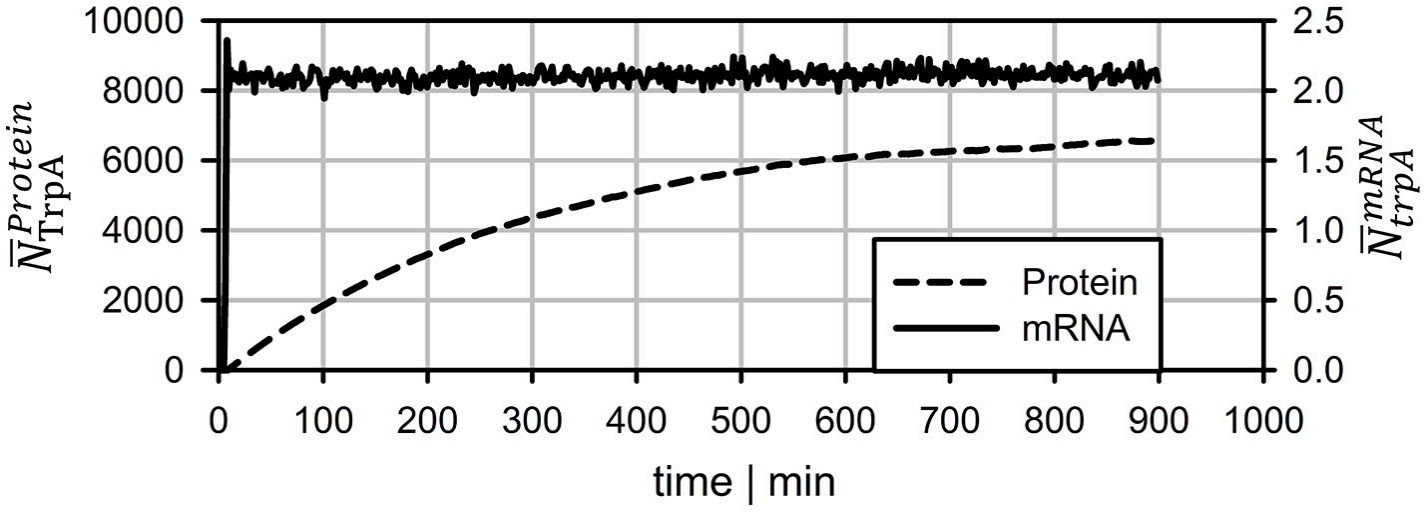
Long-term prediction of transcript and protein levels neglecting protein degradation. mRNA levels reach stable levels after ~7 min whereas protein leveling takes more than 15 h.

To compare the dynamics of transcript and protein adaptation toward new steady-states, both species were simulated. For transcript studies, the average *trpA* transcription was considered. Protein formation of TrpA disregarded putative degradation and simply considered continuous cell drain under steady-state conditions. Figure 6 clearly outlines the different speeds. Whereas, transcript levels converge to a new steady-state within 10 min (slightly more than a PFR-STR cycle), proteins need about 15 h.

## Discussion

The transcriptional dynamics observed in the STR-PFR experiments of Löffler et al. (2016) and in particular Simen et al. (2017) were modeled using a combined cell and process model. By focusing on details of transcription and translation in the cell model, a set of 10,000 individual cells was created and tracked during their repeated passages through the STR-PFR system. Inherently, the modeling approach mirrors a mechanistic understanding linking external stimuli with cellular transcriptional responses thereby excluding putative stochastic events (Elowitz et al., 2002; Avery, 2006). Accordingly, the modeling approach showed fundamental characteristics of an ensemble model, as outlined by (Henson, 2003). Here, we used the *trp* operon as an example because its polycistronic mRNA consisting of five structural genes and a leader peptide was repeatedly transcribed envisaging ammonia limitation (Simen et al., 2017) and, most importantly, its induction was followed by attenuation which directly linked transcription and translation of the gene products. Only by using the approach of ensemble modeling, individual cell fates could be tracked which finally explain the occurrence of population heterogeneity.

Comparing the experimental observations of transcript dynamics with modeling predictions, high agreement between simulations and experimental data can be observed (Figure 5). The qualification holds true not only for the prediction of short-term transcript dynamics in the PFR but also for the long-term adaptations in the STR, visualized by analyzing samples up to 28 h after initial connection of the PFR with the STR. Notably, the high precision of transcript prediction was achieved without any parameter regression. Only literature documented parameters were chosen to fix the setting of the ensemble model. Again, this finding is qualified as a confirmation of the basic approach.

Protein formation was assumed to start immediately after mRNA transcription. Unlike mRNA degradation, no distinct decay kinetics for the *trp* genes were known. Simulation studies of Figure 4 revealed that realistic protein half-lives should be about 1.1 h, which is in the range of experimental observations for other proteins (Nath and Koch, 1970; Lahtvee et al., 2014). Accordingly, impacts of protein degradation on short-term kinetics can be ruled out. However, the long-term adaptations indicated in Figure 6 are likely to be affected. The additional consideration of decay kinetics will likewise reduce steady-state levels.

One of the key findings of the STR-PFR studies of Löffler et al. (2016) and Simen et al. (2017) was the observation that PFR induced regulatory responses are propagated into STR finally causing the adaptation of the whole population. Exactly this phenomenon could be modeled as well. Figures 3, 5 document that only *trpL* and *trpE* are fully transcribed in PFR whereas the transcription of the rest of the operon continued in the STR. Subsequently, most of the stress induced cellular burden occurred after a time-delay in the well-mixed STR compartment and not immediately in the PFR, the origin of the trigger. As a consequence, the population in the STR is very heterogeneous, consisting of cells in different transcription and translation states. Some cells should be still propagating the PFR induced stress response, whereas others may have completed the same. Moreover, given that the STR and the PFR compartments do not physically exist in large-scale bioreactors, cells are expected to co-exist next to each other while circulating around. Similar studies have already be performed investigating the lifelines of fluctuating cells (Haringa et al., 2016; Kuschel et al., 2017).

## Conclusion

The ensemble model used in this study succeeded to predict experimental observations of long- and short-term transcriptional dynamics with high precision and without parameter adjustments. As such, the approach demonstrated its fundamental suitability for predicting large-scale population heterogeneity as a consequence of local stress triggers. Accordingly, likewise modeling approaches open the door for an *in silico* scale-up design, simulating large-scale performance of the cells *ab initio*.

This study illustrates that locally induced stress responses are propagated into different regions of the bioreactor thereby creating temporal and spatial inhomogeneity of the population. Notably, cellular reactions do happen on different time scales: Whereas transcriptional responses require <10 min, translational changes may continue for more than 10 h to reach new steady-states. Additionally, metabolic responses may occur which are likely to precede the transcriptional reaction. The hierarchical sequence of regulatory responses is overlaid with dynamics of mass transfer, mixing and process control which make it necessary to track individual cell responses properly for predicting large-scale performance of the total culture.

## Author contributions

AN performed the modeling, designed the study, and prepared the manuscript. ML and JS performed the experiments and prepared the manuscript. RT designed the study and prepared the manuscript.

### Conflict of interest statement

The authors declare that the research was conducted in the absence of any commercial or financial relationships that could be construed as a potential conflict of interest.
