# Supplementary material for: Repetitive Short-Term Stimuli Imposed in Poor Mixing Zones Induce Long-Term Adaptation of E. coli Cultures in Large-Scale Bioreactors: Experimental Evidence and Mathematical Model
Source: Front Microbiol. 2017 Jun 28;8:1195. doi: 10.3389/fmicb.2017.01195 (PMC5487534; doi:10.3389/fmicb.2017.01195)
Supplement: Supplementary file 1 [file Image1.PDF]

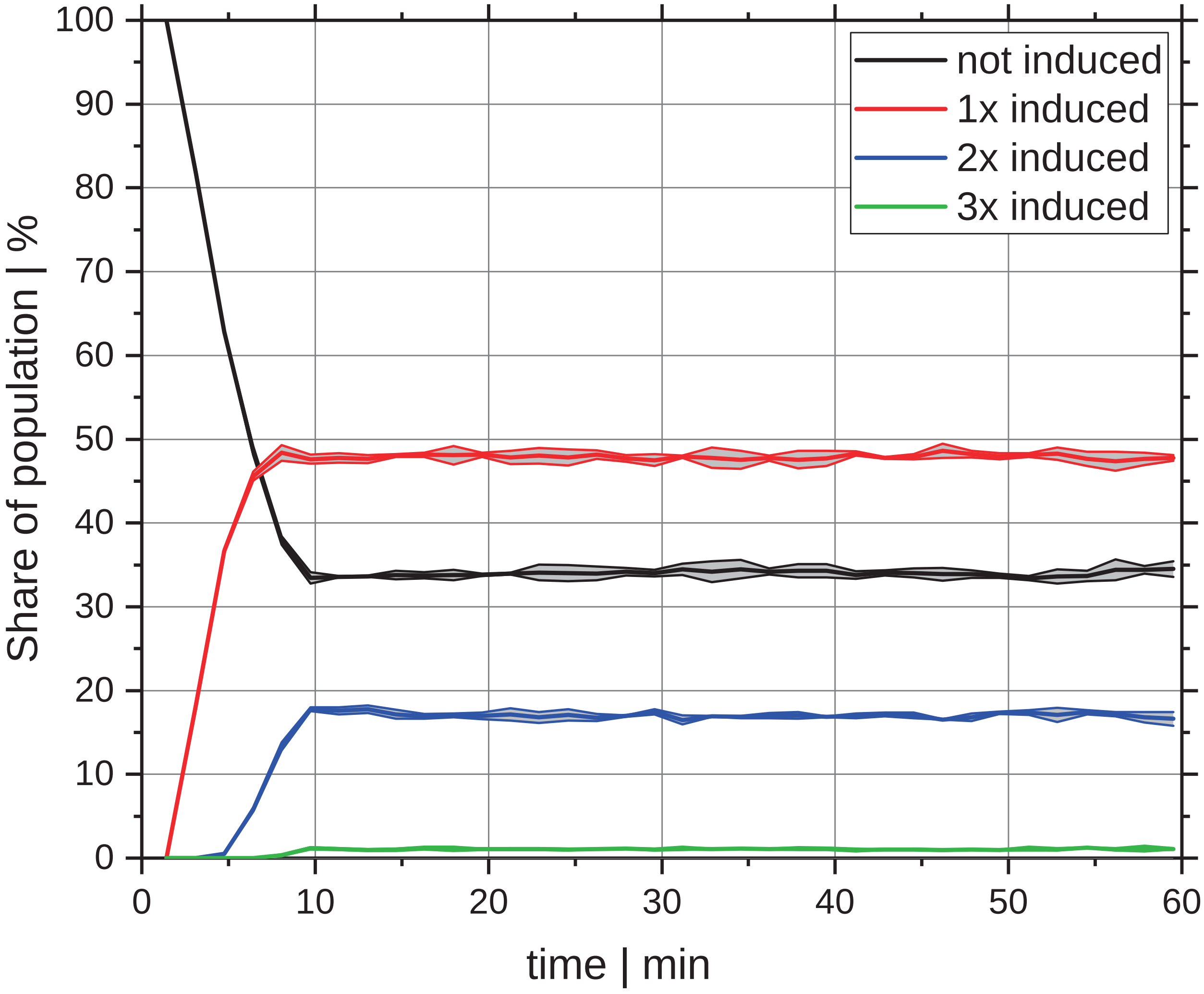

Supplementary figure 1: Time course of population distribution calculated by the ensemble model.
